# The Scent Gland Microbiomes of Wild Tamarins Provide New Insight Into Microbial Contributions to Olfactory Communication

**DOI:** 10.1002/ece3.72335

**Published:** 2025-10-30

**Authors:** Silvia Carboni, Alice C. Poirier, Ana P. Peralta‐Aguilar, Mrinalini Watsa, Gideon Erkenswick, Amanda D. Melin

**Affiliations:** ^1^ Department of Anthropology & Archaeology University of Calgary Calgary Alberta Canada; ^2^ Field Projects International Escondido California USA; ^3^ San Diego Zoo Wildlife Alliance San Diego California USA; ^4^ Department of Medical Genetics University of Calgary Calgary Alberta Canada; ^5^ Alberta Children's Hospital Research Institute University of Calgary Calgary Alberta Canada

**Keywords:** metagenomics, microbiome, olfactory communication, scent glands

## Abstract

The microbiome of mammalian scent glands is thought to contribute to the production of odorant compounds involved in sensory communication. Yet, the extent to which glandular microbiomes contain bacteria relevant to odor production and vary by host species, scent marking behavior, or gland morphology remains poorly understood, particularly in wild animals. We sampled microbes collected from skin swabs of suprapubic and sternal scent glands in wild Peruvian saddleback tamarins ( *Leontocebus weddelli* ; *n* = 19) and emperor tamarins (*Tamarinus imperator*; *n* = 20) to better understand glandular microbial communities. We aimed to: (1) profile glandular microbiomes of both species, focusing on odor‐related taxa and metabolic pathways, and (2) determine whether suprapubic glands, more often in contact with the external environment, had higher diversity and distinct composition of odor‐related taxa and pathways compared to sternal glands. We generated metagenomic reads using short‐read DNA shotgun sequencing from glandular swabs. We identified 18 odor‐associated microbial taxa in both tamarin species, mainly *Staphylococcus* and *Corynebacterium*, and 26 pathways, including pyruvate fermentation and amino acid metabolism. Suprapubic glands had lower Shannon alpha diversity relative to sternal glands, especially in *L. weddelli* . The glands of *L. weddelli* also differed in taxonomic composition, with odor‐related taxa more abundant in suprapubic glands. Our results provide evidence for the involvement of scent gland microbiomes in host communication biology. Glandular specializations differed not only between closely related tamarin species but also between gland types within the same individuals, suggesting a nuanced pattern of host–microbe coevolution that may shape interactions important for olfactory communication.

## Graphical abstract

Our study investigated the microbiomes of suprapubic and sternal scent glands in two wild tamarin species to explore their putative roles in odor production and communication. Using metagenomic sequencing, we identified 18 odor‐associated microbial taxa and 26 related metabolic pathways, finding significant differences in microbial diversity and composition between gland types and host species. These findings suggest that scent gland microbiomes contribute to olfactory communication and reflect a complex pattern of host–microbe coevolution.

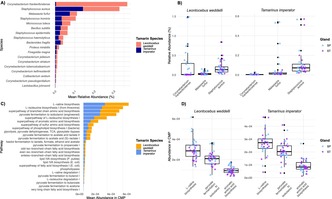

## 1 Introduction

Microbial communities colonize seemingly every part of living animals (Zilber‐Rosenberg and Rosenberg 2008). The skin, the largest existing organ, is increasingly recognized as hosting a highly variable microbiome, both throughout the host's lifespan and across different regions of the body (Luna 2020). Distinct skin regions harbor microbial communities with differing structures, compositions, and functions. Intriguingly, a growing body of evidence suggests that the microbiome inhabiting mammalian scent glands contributes meaningfully to the production of body odor used for olfactory communication (Carthey et al. 2018; Ezenwa and Williams 2014). Olfaction is one of the most widespread modalities of communication, underpinning many social and reproductive behaviors, such as individual recognition, territory defense, and the assessment of competitive, genetic, and reproductive characteristics in many mammals, including humans (Drea 2015; Drea et al. 2002; Miller et al. 2003; Rich and Hurst 1998; Setchell et al. 2010).

The fermentation hypothesis provides a compelling framework for investigating the contributions of microbes to mammalian scents and thereby their interactions with one another (Carthey et al. 2018; Ezenwa and Williams 2014). According to this hypothesis, scent glands provide a nutrient‐rich habitat for microbes, which process sebum and other substances found in glandular secretions as part of their metabolism (Carthey et al. 2018). In doing so, microbes release volatile organic compounds (VOCs) as by‐products, and thereby contribute to the body odor of the host (Hara et al. 2014; James et al. 2004; James et al. 2013). As a result, the fermentation hypothesis predicts that changes in the glandular microbiome lead to a shift in the VOCs released, resulting in the covariation between the compositions of odor and glandular microbiome (Archie and Theis 2011; Council et al. 2016; Ezenwa and Williams 2014).

A first step towards understanding the proximate mechanisms underlying microbial contributions to olfactory communication is to describe a comprehensive microbial profile for each type of gland surface. Bacterial genera such as *Bacillus*, *Staphylococcus*, *Aerococcus*, *Anaerococcus*, *Clostridium*, *Lactobacillus*, *Corynebacterium*, *Proteus*, and *Bacteroides*, belonging to the phyla *F* *irmicutes*, *Actinobacteria*, *Proteobacteria*, and *Bacteroidetes*, are abundant members of the glandular microbiota of mammals, including humans and non‐human primates, suggesting that some may contribute to odor production (Bowen et al. 2021; Greene et al. 2019; Leclaire et al. 2017; Ma et al. 2022; Rojas et al. 2023; Sin et al. 2012; Theis et al. 2013; Troccaz et al. 2015). Researchers studying wild lemurs, hyenas, and meerkats, as well as captive pandas and domestic cats, have found significant covariation between VOCs from glandular secretions and glandular microbiota (Greene et al. 2019; Leclaire et al. 2017; Ma et al. 2022; Rojas et al. 2023; Theis et al. 2013). These studies collectively offer valuable insights into the presence of bacteria in glandular areas, but often overlook other key microbial taxonomic domains (e.g., eukaryotes such as yeast) that may also contribute to the structure of skin microbiomes. With the continuous advancement of sequencing technologies and bioinformatics tools, the glandular microbiome can more easily be characterized, allowing us to assess the functional roles of these microbes and identify pathways potentially involved in odor production and olfactory communication.

Understanding the extent of microbial contributions to olfactory communication also requires delving into microbial fermentation processes and other metabolic pathways that produce VOCs as by‐products. Studies of the chemical origin of odor suggest that microbes play a key role in producing fatty acids, amines, sulfur compounds, alcohols, and esters (Hara et al. 2014; James et al. 2004; Lam et al. 2018; Zhou et al. 2021). The main metabolic pathways responsible for producing these compounds include the metabolism of branched‐chain amino acids (e.g., leucine, valine, and isoleucine) and aromatic amino acids, fatty acid biosynthesis, lipid and pyruvate metabolism, and the synthesis of ketones and steroids, primarily carried out by the bacterial genera *Cutibacterium*, *Staphylococcus*, and *Corynebacterium* (Hara et al. 2014; James et al. 2013; Rojas et al. 2023; Troccaz et al. 2015). A recent study also reported the importance of yeast (*Malassezia furfur*) in producing volatile compounds (Gonzalez et al. 2019).

Research on microbiomes and their putative functions in wild environments reveals the ecological factors that shape the evolution of the microbe‐olfaction axis, identifying key variables that may impact olfactory communication in different species. In captive settings, these variables may be masked by artificial factors known to alter microbiomes, as observed in comparative studies of the gut microbiota (Greene et al. 2019; Bensch et al. 2023; Wang et al. 2023) and the skin microbiota (Ross et al. 2019) of captive and wild populations. Although less studied, scent gland microbiota also differ between captive and wild settings in species such as the giant panda ( *Ailuropoda melanoleuca* ) (Zhou et al. 2021) and lemurs (*Propithecus* spp., *Eulemur* spp., and *Lemur catta* ) (Greene et al. 2019). These microbial variations may influence odor composition and ultimately patterns of olfactory communication in wild contexts, which are invaluable for understanding the role of microbiota in olfactory communication.

**FIGURE 1:**
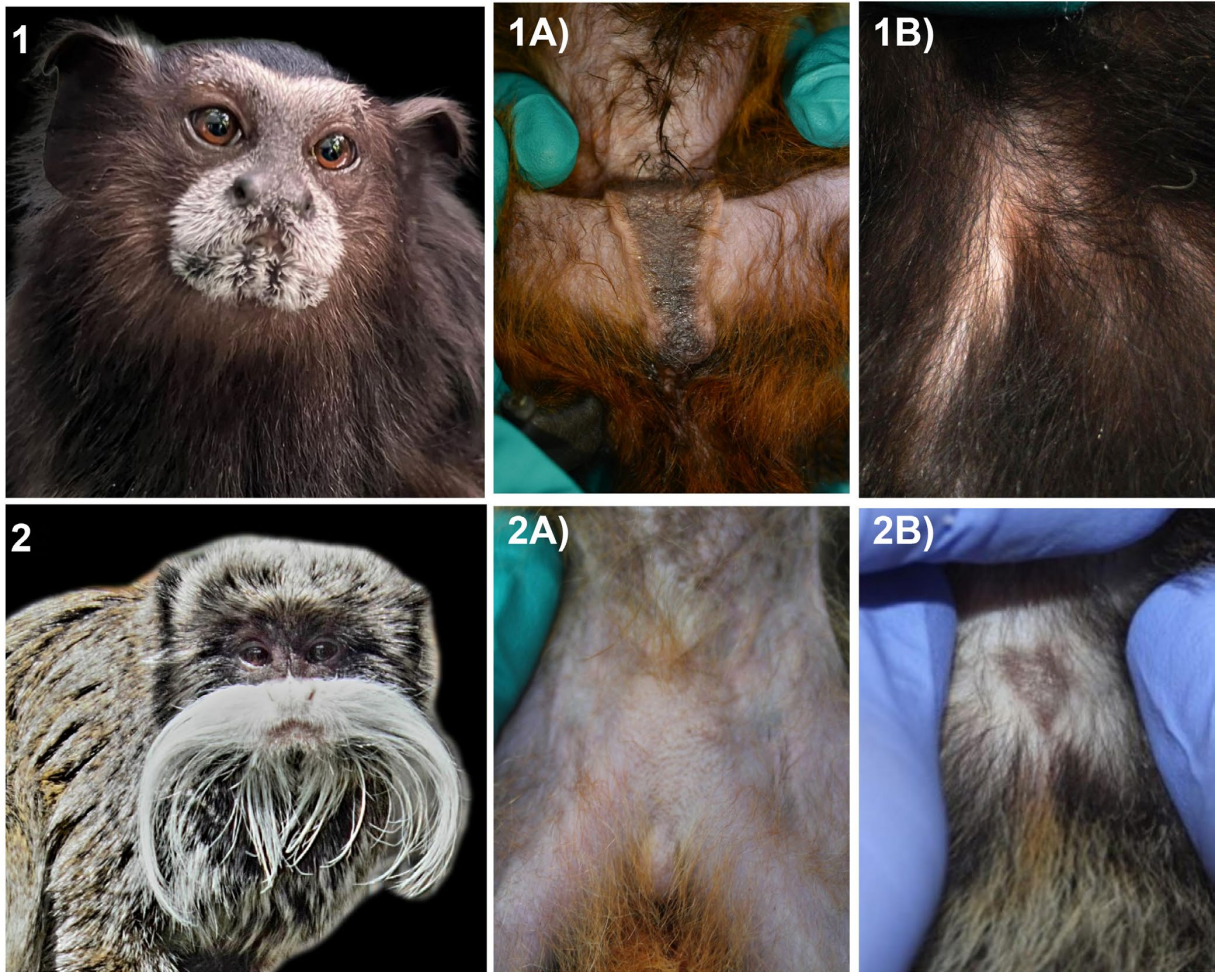
Pictures of scent glands of *L. weddelli* and *T. imperator* . (1) Adult female *Leontocebus wedelli*'s (a) suprapubic gland, and (b) sternal gland. (2) Adult female *Tamarinus imperator*'s (a) suprapubic gland, and (b) sternal gland.

Many mammals have specialized scent glands for depositing odors onto substrates (Mykytowycz and Goodrich 1974; Quay 1986). Although primates are often characterized as reliant on vision, growing evidence suggests that olfaction also plays a significant role in social and reproductive communication among primates (Poirier and Melin 2024). Indeed, many monkeys in the Americas (Parvorder: Platyrrhini) have specialized morphology and behavior for olfactory communication (Heymann 2022), and are great models to test predictions of the fermentation hypothesis. Among them, tamarins have three specialized scent glands (Figure 1)—a sternal gland, a suprapubic gland, and an anal gland—that are involved in scent marking behavior, that is, the act of conspicuously depositing glandular secretions on a surface (Heymann 2006). Within tamarin species, sternal glands are usually the smallest, but the histology of all three is similar with abundant sebaceous and apocrine glands in the dermis (Moraes et al. 2006). All three glands are typically rubbed on branches, lianas, and stems in the act of scent marking (Heymann 2001; Heymann 2006; Miller et al. 2003). The use of the suprapubic and anogenital glands, either separately or in combination (i.e., circumgenital scent marking), is more common than the use of the sternal gland, which is typically the least frequently used in callitrichids (i.e., marmosets and tamarins) (Heymann 2001; Heymann 2022; Poirier et al. 2021a). The frequency of gland use is relevant as environmental rubbing may favor microbial exchange with surroundings (Bowen et al. 2021; Sarkar et al. 2020). Therefore, the microbiota found on the surface of scent glands is ultimately a combination of environmentally acquired microbes and those specific to the individual, and possibly to the gland itself (Bowen et al. 2021; Ezenwa and Williams 2014).

Here, we study two sympatric species of Callitrichidae wild tamarins (saddleback tamarins— *Leontocebus weddelli* , *n* = 19; and emperor tamarins—*Tamarinus imperator*, *n* = 20) living in tropical lowland forests of Peru. Our two main aims are: (1) to profile the taxonomy and putative functional capacity of microbes in the sternal and suprapubic glands, to then be able to (2) test predictions stemming from the fermentation hypothesis. Specifically, that (a) microbes linked to volatile odor production will be prevalent in these glands, and that (b) there will be a greater microbial diversity and increased abundance of microbes and metabolic pathways linked to odor production in the suprapubic glands of both species, which are more frequently used during scent marking compared to the sternal glands. Given that tamarin species vary in scent marking frequencies and behaviors (Heymann 2001), as well as in the pigmentation of their scent glands, darker and easier to spot in *L. weddelli* (Figure 1), we also explore differences in the gland morphology between the two species, and we controlled for species‐level differences in our analysis.

## 2 Methods

### 2.1 Study Site and Species

We studied wild saddleback tamarins ( *Leontocebus weddelli* ) and emperor tamarins (*Tamarinus imperator*) at the Estación Biológica Río Los Amigos (EBLA) in the Madre de Dios Department of southeast Peru (12°34′07″ S, 70°05′57″ W). The 5 km^2^ field station is a biodiverse, protected conservation area managed by Conservación Amazónica‐ACCA and is located between the Los Amigos and Madre de Dios rivers. The wet season lasts from October to March, while the dry season extends from April to September (Watsa 2013). Average temperatures remain relatively stable year‐round, typically ranging from 21°C to 25°C. However, the region is also prone to “*friajes*”—cold surges that occur periodically during the driest months, causing a rapid temperature drop lasting between 3 and 7 days.

Saddleback and emperor tamarins are sympatric at EBLA, living in groups of 3 to 9 individuals, and displaying similar social and breeding systems (Goldizen et al. 1996; Watsa 2013). Both species are polyandrous, typically forming groups consisting of one breeding female, her offspring, multiple males, and usually one or two non‐breeding females (Watsa 2013). Crucially, both species possess morphological adaptations (e.g., well‐developed scent glands and a functional vomeronasal organ) and behavioral traits (e.g., scent‐marking behaviors) that facilitate olfactory communication (Heymann 2006).

### 2.2 Sample Collection

Between June and July 2022, we sampled 6 groups of *L. weddelli* (*n* = 19 individuals) and 6 groups of *T. imperator* (*n* = 20 individuals). The tamarin population is part of a long‐term annual mark‐recapture program conducted by Field Projects International (FPI) (Watsa et al. 2023). The protocol involves careful planning of trapping events following habituation and observation of the group at multi‐compartment traps, as well as the use of low doses of anesthetics. The full methodology is described in detail in Watsa et al. (2023). Animals were individually identified using subcutaneous microchips, and age class (adult, sub‐adult, juvenile) was determined based on the year of birth and dental eruption patterns (Watsa 2013; Watsa et al. 2023). From each adult animal, we measured the length and width of both scent glands using a caliper (in mm), and recorded the body weight using a scale with a maximum capacity of 1 kg. Gland size measurements were not collected from sub‐adult and juvenile animals due to their underdeveloped state and the difficulty in accurately identifying the glands with the naked eye. To collect microbiome samples, we swabbed the glandular surface (hereafter, glandular samples) of all the captured individuals for 10 s using dry sterile swabs (BD BBL CultureSwab). One swab per animal was used for the suprapubic gland, and one for the sternal gland. We collected only one suprapubic swab and one sternal swab per animal, as analysis of the anal glands is challenged by exposure to fecal and genital microbes. We chose to use dry swabs over moist ones to facilitate the collection of microbes that cannot adhere to wet surfaces (Manus et al. 2022). Although this method samples only the gland surface, it is minimally invasive and fast, key advantages for sampling wild animals, and allows direct comparison across individuals, scent‐marking use, and environmental exposure. To help control for environmental contamination, we also collected an ambient control on each trapping day (*n* = 11) by exposing a clean swab to the air for 10 s. After swabbing, we immediately placed the swabs in their sterile plastic pouch on ice, and then stored them at−25°C within 6 h. In total, we collected 39 suprapubic samples ( *L. weddelli* *n* = 19; *T. imperator* *n* = 20) and 37 sternal samples ( *L. weddelli* *n* = 18; *T. imperator* *n* = 19), as the latter could not be collected from one individual of each tamarin species (Table S1).

This study followed the guidelines of the Canadian Council of Animal Care and was approved by the Animal Care Committees of the University of Calgary (ACC19‐0167) and Washington University in St. Louis (#21‐0084). The annual mark‐recapture program and the sample collection conducted by FPI are authorized by the Peruvian Ministry of the Environment (SERFOR‐D000116‐2021).

### 2.3 Microbiome Analysis

We extracted and purified the DNA from each sample using the DNeasy PowerSoil Pro Kit with spin column (Qiagen ID 47017) in the Los Amigos Wildlife Conservation Laboratory, a BSL‐2 genetic lab located on site (In Situ Lab Initiative), and stored at −25°C. All the samples were extracted within a month of their collection date, under sterile conditions in biosafety hoods. We followed the manufacturer's protocol, but skipped the steps with CD2 buffer to prevent excess DNA loss in our low‐concentration samples. This step is primarily designed to remove inhibitors and is especially critical for fecal samples. We included seven laboratory extraction controls following best practices in microbiome research (Eisenhofer et al. 2019; Kim et al. 2017). DNA samples were exported to the University of Calgary, on ice. The Center for Health Genomics and Informatics prepared metagenomic libraries per sample using the NEB Ultra II DNA library prep kit and sequenced them on an Illumina NovaSeqX, PE × 100 bp.

We processed the sequencing data using the software tools Seqkit, FastQC, and MultiQC (Ewels et al. 2016; Shen et al. 2016), to assess sample quality and verify that sequencing was successful for all samples. Average yield per sample was 3 Mb (±0.4). We then polished the raw reads from the adaptors, and we excluded all reads shorter than 50 bp with Trimmomatic (Bolger et al. 2014). After this step, excluding the controls from the dataset, we obtained an average of 12,629,077 (±1,839,884) metagenomic pair‐end reads per sample (minimum = 6,709,113; maximum = 17,174,164). We then removed the host reads using Bowtie2 (Langmead and Salzberg 2012), by aligning the reads to the host genome from NCBI (GCA_004024885.1_SagImp_v1_BIUU_genomic.fna) and removing the overlapping reads. On this cleaned dataset, we performed taxonomic assignment using Kaiju, a tool that employs protein‐level, high‐sensitivity classification (Menzel et al. 2016). Kaiju uses reference databases derived from NCBI, including protein sequences and taxonomy files. In our analysis, we used the pre‐built Kaiju database nr_euk_2023‐05‐10, which includes archaea, bacteria, viruses, and microbial eukaryotes. Given the high incidence of false positives with Kaiju, we selected the maximum exact matches (MEMs) mode to improve precision (Menzel et al. 2016). To investigate functional capacity, we used Humann3.6 to process the concatenated forward and reverse reads, allowing us to identify the abundance of each identified metabolic pathway (Beghini et al. 2021). Using this tool, we performed functional annotation by mapping reads identified with MetaPhlAn and the CHOCOPhlAn database to species‐specific pangenomes, and aligning unclassified translated reads against the UniRef50 protein database using DIAMOND to assign gene families. These gene families were subsequently mapped to metabolic pathways using the MetaCyc database to estimate pathway abundances. The output, initially in RPK (reads per kilobase), was normalized to CPM (copies per million), which accounts for differences in the total number of counts per sample. The final output consisted of two datasets: one reflecting taxonomic abundance and another for metabolic pathways, which we used for subsequent analysis.

Using R software (R Core Team 2024), we used the package decontam in prevalence mode (threshold 0.1) (Davis et al. 2018) to identify and remove contaminants from the field and laboratory controls. Additionally, we only kept the taxa present in more than 10 samples and with relative abundance higher than 0.001%, as a threshold to improve the precision and sensitivity of Kaiju and optimize the classifier performance (Edwin et al. 2024). We then used the package phyloseq (McMurdie and Holmes 2013) to calculate two measures of alpha diversity from taxonomic abundances: (1) the Chao1 index, which estimates richness (i.e., the number of species in a sample) by accounting for undetected taxa, based on the number of rare species—specifically the singletons and doubletons of a sample, and (2) the Shannon index, which estimates both richness and evenness (i.e., if the community is dominated by few species or if they are equally distributed). Similarly, we calculated richness and the Shannon index for the functional capacity dataset with the package vegan (Oksanen et al. 2024). We also used vegan to estimate beta diversity by calculating the robust Aitchison distance metric from center‐log transformed taxonomic data and CPM (copies per million) transformed pathway abundances, to account for the compositional nature of microbiome data (Gloor et al. 2017; Martino et al. 2019).

To identify bacteria associated with odor production, we performed a systematic literature search using combinations of keywords related to microbes and odor. Search terms included “bacteria,” “microbe,” “microbiome,” and “microbiota,” combined with odor‐related terms such as “odor,” “odor,” “smell,” and “volatile organic compound.” We screened studies to select those that provided experimental evidence linking microbes to the production of volatile organic compounds (VOCs), focusing on culture‐based experiments and metabolomics analyses. From these studies, we compiled a list of microbial taxa known to inhabit the glandular skin of mammals and demonstrated to contribute to odor formation.

### 2.4 Statistical Analysis

We conducted all statistical analysis in R. We visualized the composition of microbial communities in the two scent glands of the two tamarin species (Aim 1) using bar plots generated with ggplot2 (Wickham 2009) to display the relative and total abundances of taxa and pathways. We then described the distribution of taxa and pathways that are linked to odor production (Aim 2a) by visualizing their mean abundance across all samples.

For each gland type, we characterized interspecies differences in the host by conducting Wilcoxon tests comparing the ratios of gland length and width to body weight between the two species. We then tested if, within species, the two types of scent glands differed in diversity and composition of taxa and metabolic pathways to reflect their different use in scent marking behavior (Aim 2b). To do so, we first fitted general linear mixed models (GLMMs) using the glmer function from the lme4 package. We built three competing models for each response variable (i.e., Shannon index and Chao1 index) and assessed the best performing models by comparing AIC values. Model 1 only included the interaction between tamarin species and gland types as a fixed effect; models 2 and 3 also included the effect of either sex and age class, respectively. Sex and age were compared separately to avoid overfitting. In all models, we incorporated individual ID, nested with group ID, as a random effect to control for sampling different body parts of the same individual and for the potential sharing of microbes among group members in close contact. We chose a Gamma distribution with a log‐link function to follow the right‐skew distribution of our continuous data. We then tested for multicollinearity by calculating the Variance Inflation Factor, and we assessed autocorrelation in the residuals using the Durbin‐Watson test. The results of model comparison are presented in Table S2. In all models, there was no multicollinearity, and the Durbin‐Watson test was close to 2, indicating no significant autocorrelation. In this study, we chose not to apply rarefaction in order to avoid discarding potentially valuable data. Instead, we verified that our results were not influenced by read count. To do so, when a predictor variable was significant with respect to the response variable, we ran the same model with the residuals of the alpha diversity index (i.e., the portion of alpha diversity unexplained by read count) as the new response variable and we verified that this residual model was still significant. We also conducted a PERMANOVA using the vegan package to assess the effect of the interaction between gland type and tamarin species, as well as the influence of age and sex, on Aitchison distance. To account for potential social group structure, we grouped the data by social group and included it as a stratification factor in the analysis. Finally, to explore whether the two glands differed in the abundance of taxa or metabolic pathways related to odor production, we separated the databases of *L. weddelli* and *T. imperator* , and we used the ancombc2 package to perform ANCOM‐BC tests on the two gland types. We chose the more conservative “Holm” method to control for false positives.

## 3 Results

### 3.1 Taxonomy and Functional Capacity of Glandular Microbiota

**FIGURE 2:**
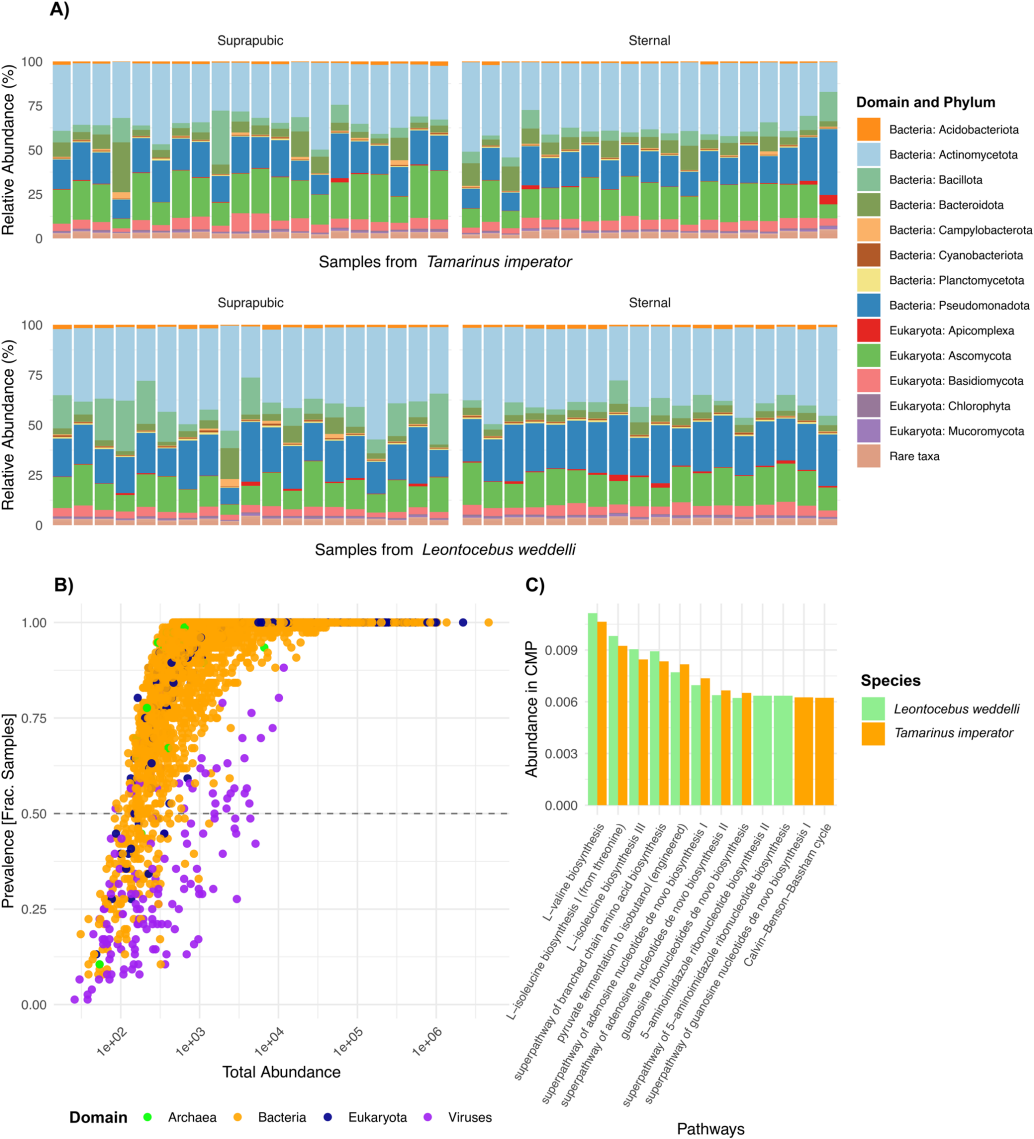
Profile of the glandular microbiome, through representing the taxonomy and metabolic pathways found in the two tamarin species. Panel (A) displays the proportion of each phylum relative to the total abundance of all taxa in samples from *T. imperator* (*n* = 39) and *L. weddelli* (*n* = 37) separated by gland type. The legend only shows the 13 phyla with mean abundance > 0.005, while the rest are considered “Rare taxa”. Panel (B) represents the relationship between species prevalence and their total abundance in the dataset. The prevalence is the fraction of samples in which a species is present. Each point represents a species, and the color corresponds to its taxonomic Domain. The horizontal dashed line at 0.5 prevalence indicates the threshold for species that are present in at least half of the samples. Panel (C) shows the total abundance in copies per million (CPM) of the ten most abundant metabolic pathways in the microbiome samples of *T. imperator* and *L. weddelli* . In panels (B, C), the total abundance of a taxon or a pathway is the sum of its abundances across all samples.

**FIGURE 3:**
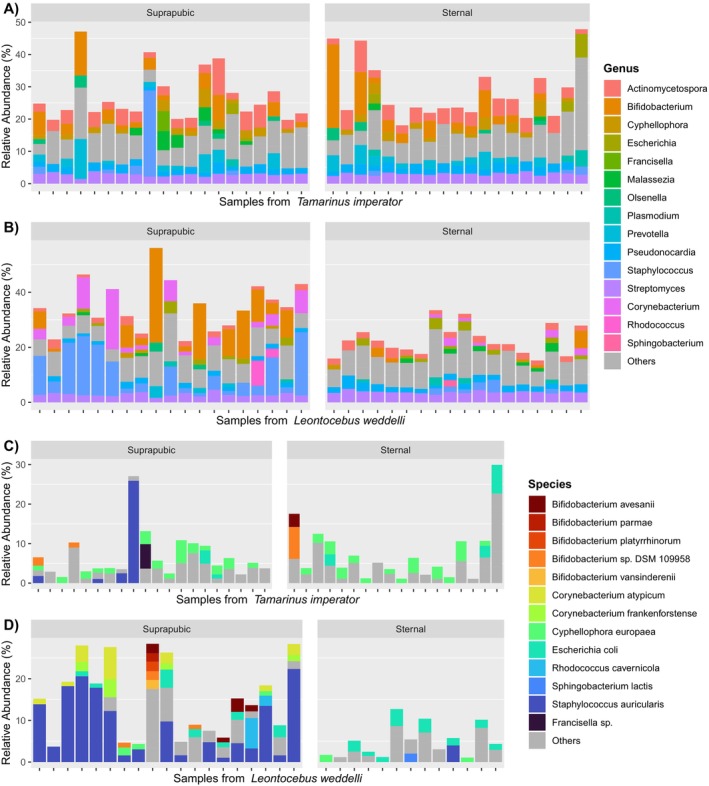
Microbial genera and species with relative abundance > 1%. Panels (A, B) depict the genera in each sample of *T. imperator* (A) and *L. weddelli* (B). Panels (C, D) represent the species in each sample of *T. imperator* (C) and *L. weddelli* (D). All four graphs are separated by gland type. The two legends show only the taxa with mean abundance > 0.02, while the rest are grouped under the name “Others”.

At the taxonomic Domain level, *Bacteria* represented 74.5% of the microbial community in our samples, while *Eukaryota* represented 25.0%. This included the bacterial phyla *Actinomycetota* (i.e., *Actinobacteria*), which represented 37.3% of the total phyla abundance, followed by *Pseudomonadota* (i.e., *Proteobacteria*) (19.0%), *Bacillota* (i.e., *Firmicutes*) (7.6%), and *Bacteroidota* (5.8%), and the eukaryotic phyla *Ascomycota* (17.4%) (Figure 2A,B). However, *L. weddelli* presented relatively more *Bacillota* (9.14%) compared to *T. imperator* (6.25%) (Figure 2A). At the genus level, both tamarin species showed a high prevalence of the bacterial genera *Bifidobacterium* (3.84%), *Staphylococcus* (3.13%), *Actinomycetospora* (3.11%), *Streptomyces* (3.03%), *Pseudonocardia* (2.07%), and *Sphingomonas* (1.73%), with higher representation of *Staphylococcus* in *L. weddelli* (5.34%) and of *Actinomycetospora* in *T. imperator* (4.10%) (Figure 3A,B). We then calculated relative abundances only within the bacterial subset, excluding other microbes. The representation of these genera was found to be only modestly higher, indicating that their relative contributions to the community were largely maintained. Interestingly, at the primate species level, *L. weddelli's* microbial community was highly represented by the bacterium *Staphylococcus auricularis* (4.76%), while *T. imperator* was relatively rich in the fungus *Cyphellophora europaea* (1.82%) (Figure 3C,D).

We found that the most abundant metabolic pathways predicted in our samples were primarily involved in amino acid and nucleotide biosynthesis, energy production, and cellular metabolism. These pathways were largely shared between the two tamarin species, including those related to fermentation processes, such as L‐valine and L‐isoleucine biosynthesis, the super‐pathway of branched‐chain amino acid biosynthesis, and pyruvate fermentation (Figure 2C).

### 3.2 Glandular Microbes That Might Contribute to Odor Production

**FIGURE 4:**
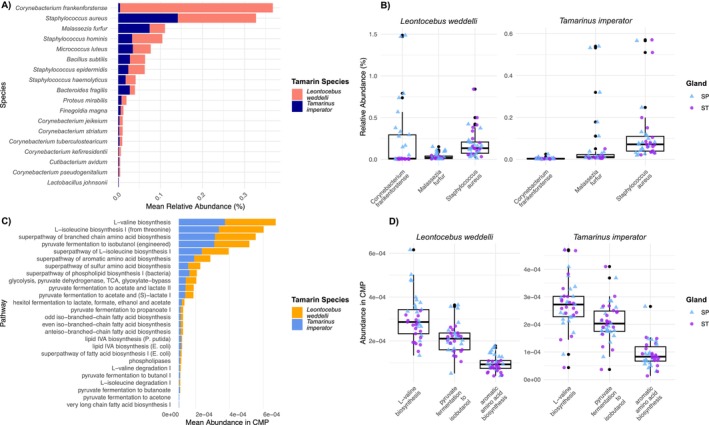
Relative abundance of microbial taxa and metabolic pathways potentially involved in the synthesis of VOCs. Panel (A) displays the mean relative abundance in percentage (on the left) of 18 microbial species found in glandular samples of *L. weddelli* and *T. imperator* that are potentially involved in odor production. On Panel (B), the three microbial species with the highest mean relative abundance across all samples are separated by tamarin species, to show the percentage of their relative abundance in each sample per gland type. The y‐axis limits have been set to focus on the majority of data points, which may exclude the highest values, especially for the *Corynebacterium frankenforstense* in suprapubic glands of *L. weddelli* . Panel (C) displays the mean abundance in copies per million (CPM) of 26 metabolic pathways that are potentially involved in odor production in *L. weddelli* and *T. imperator* , while Panel (D) focuses on the three metabolic pathways with the highest mean abundance, showing their abundance in each sample, separated by tamarin species and gland type.

From the list of microbes associated with odor production compiled through literature search, we found 18 microbial taxa in the glandular samples of both tamarin species, potentially associated with odor production (Table 1). The genera with higher abundance relative to the total abundance of all genera in the microbial community across all samples were the bacteria *Staphylococcus* (3.13%), *Streptococcus* (0.456%), and *Lactobacillus* (0.124%), all belonging to the phylum *Bacillota*; *Corynebacterium* (1.49%), *Micrococcus* (0.230%), and *Cutibacterium* (0.134%), as part of the *Actinomycetota* phylum; *Pseudomonas* (0.824%) from the *Pseudomonadota*, and *Bacteroides* (0.583%), from the *Bacteroidota* phylum. Among the eukaryotes, the genus *Malassezia* contributed 0.863% to the overall microbial composition. At the species level, the bacteria *Corynebacterium frankenforstense*, *Staphylococcus aureus* , and the fungus *Malassezia furfur* exhibited the highest mean relative abundances across all samples (Figure 4A,B).

**TABLE 1:** List of microbial species reported to be associated with odor production, along with their respective phylum and the percentage contribution of each taxon to the total abundance across all taxa in the datasets of *Leontocebus weddelli* (LWED; *n* = 37) and *Tamarinus imperator* (TIMP; *n* = 39). References to relevant literature on the microbial species and their putative influence on body odor are also included.

Phylum	Species	%LWED	%TIMP	Literature
*Bacillota*	*Staphylococcus epidermidis*	0.019	0.013	Hara et al. (2014), Lam et al. (2018), Starkenmann et al. (2005)
*Bacillota*	*Staphylococcus hominis*	0.036	0.017	Lam et al. (2018), Troccaz et al. (2015), Zinn et al. (2021)
*Bacillota*	*Staphylococcus aureus*	0.081	0.073	Hara et al. (2014)
*Bacillota*	*Staphylococcus haemolyticus*	0.011	0.009	Starkenmann et al. (2005)
*Bacillota*	*Bacillus subtilis*	0.015	0.014	Gao et al. (2018)
*Bacillota*	*Finegoldia magna*	0.003	0.003	Yamaguchi et al. (2019)
*Bacillota*	*Lactobacillus johnsonii*	2.4e‐04	4.3e‐04	Rojas et al. (2023)
*Actinomycetota*	*Corynebacterium frankenforstense*	0.193	0.002	Rojas et al. (2023)
*Actinomycetota*	*Corynebacterium pseudogenitalium*	0.001	7.7e‐04	Lam et al. (2018)
*Actinomycetota*	*Corynebacterium tuberculostearicum*	0.003	0.002	James et al. (2013), Lam et al. (2018), Troccaz et al. (2015)
*Actinomycetota*	*Corynebacterium striatum*	0.004	0.001	Natsch et al. (2003)
*Actinomycetota*	*Corynebacterium jeikeium*	0.004	0.001	Zinn et al. (2021)
*Actinomycetota*	*Corynebacterium kefirresidentii*	0.002	6.3e‐04	Swaney et al. (2023)
*Actinomycetota*	*Micrococcus luteus*	0.020	0.019	Zinn et al. (2021)
*Actinomycetota*	*Cutibacterium avidum*	0.001	9.3e‐04	Lam et al. (2018)
*Pseudomonadota*	*Proteus mirabilis*	0.004	0.004	Nordstrom et al. (1989), Rojas et al. (2023)
*Bacteroidota*	*Bacteroides fragilis*	0.005	0.016	Rojas et al. (2023), Yamaguchi et al. (2019)
*Basidiomycota*	*Malassezia furfur*	0.017	0.046	Gonzalez et al. (2019)

We identified the presence of metabolic pathways with a putative involvement in odor production. Among the most abundant in our dataset were pathways involved in pyruvate metabolism and fermentation, in aromatic amino acid biosynthesis, and in branched‐chain amino acid metabolism, specifically of valine and isoleucine (Figure 4C,D).

### 3.3 Difference in Diversity and Composition of Microbial Taxa and Metabolic Pathways Between the Two Gland Types

In our population, interspecific differences in gland size were only significant for suprapubic glands. When looking at the two sexes separately, the effect was only significant in males. Adult *L. weddelli* had longer and wider suprapubic glands relative to body weight than adult *T. imperator* (Length: *W* = 115.5, *p*‐value = 0.01; Width: *W* = 109, *p*‐value = 0.03), while there was no difference in the measures of the sternal glands (Length: *W* = 48, *p*‐value = 1; Width: *W* = 71, *p*‐value = 0.083) (Table S3, Figure S1).

**FIGURE 5:**
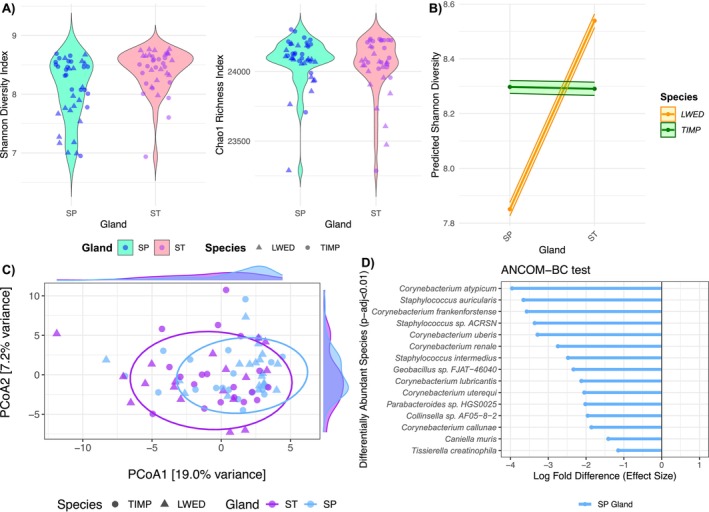
(A) Values of Shannon diversity index and Chao1 richness index calculated in suprapubic (SP) and sternal (ST) glands of both tamarin species (LWED and TIMP). (B) Predicted values of Shannon diversity based on the fitted GLMM model for the interaction effect between gland type and tamarin species. The two points represent the model‐predicted mean Shannon diversity values for each combination. Sternal glands were more diverse than suprapubic glands, and the effect was more pronounced in *L. weddelli* . (C) The Principal Coordinates Analysis (PCoA) calculated using the robust Aitchison distance, showed that the *x*‐axis (PCoA1) and *y*‐axis (PCoA2) explain 19% and 7.2% of the variation in microbial community composition, respectively. The ellipses represent the 85% confidence intervals for each gland type, providing an indication of the clustering and dispersion of samples within each group. The density plots on the axes represent the microbial communities across the gland types. (D) Differential abundance of taxa tested with ANCOM‐BC by gland type in *L. weddelli* . All taxa shown are depleted in the sternal gland compared to suprapubic glands.

We first tested for putative differences in taxonomic diversity measures between the two gland types. While there was no effect of tamarin species and gland type on the Chao1 index (Species: Estimate = 0.005, SE = 0.003, *z* = 1.824, *p* = 0.068; Gland: Estimate = −0.002, SE = 0.002, *z* = −0.899, *p* = 0.369), sternal glands had higher Shannon diversity compared to suprapubic glands, although the magnitude of the difference was small (Estimate = 0.084, SE = 0.015, *z* = 5.49, *p* < 0.01). The difference was significant only in *L. weddelli* (Interaction: Estimate = −0.085, SE = 0.021, *z* = −3.977, *p* < 0.01) (Figure 5A,B). The residual model supported this result (Interaction: Estimate = −0.668, SE = 0.196, *z* = −3.413, *p* < 0.01). When testing for differences in beta diversity, the interaction between tamarin species and gland type was significant (Interaction: F = 1.605, R^2^ = 0.019, *p* = 0.031) (Figure 5C). Additionally, we found an effect of age class (F = 1.904, R^2^ = 0.045, *p* < 0.01), but not of sex (*F* = 0.999, *R* ^2^ = 0.012, *p* = 0.446). The residual variance was substantial (*R* ^2^ = 0.817), indicating that the fixed effects explained less than 20% of the variation in community structure, possibly suggesting high interindividual variation.

Microbial communities of the two glands differed for the abundance of several taxa only in *L. weddelli* . In this species, 15 microbial taxa showed decreased abundance in the sternal glands compared to the suprapubic ones (Figure 5D). All the results passed the sensitivity analysis, which checks for false positives (Table S4). In *T. imperator* , there was no significant difference in the abundance of taxa between sternal and suprapubic glands.

The distribution of metabolic functions in the sternal and suprapubic glands showed substantial overlap in both tamarin species, with generally high abundances of L‐valine biosynthesis and pyruvate fermentation pathways. We observed only small variations in the mean abundances across pathways related to amino acid and nucleotide biosynthesis and glycolysis (Figure S2).

We found no significant effect of gland type nor of tamarin species on either the Shannon index (Species: Estimate = 0.005, SE = 0.007, *z* = 0.710, *p* = 0.478; Gland: Estimate = 0.007, SE = 0.005, *z* = 1.285, *p* = 0.199) or the richness of putative metabolic pathways (Species: Estimate = 0.001, SE = 0.057, *z* = 0.014, *p* = 0.989; Gland: Estimate = −0.013, SE = 0.044, *z* = −0.300, *p* = 0.764) (Figure S3), and the models did not outperform the respective null models (Table S2). Additionally, our predictor variables did not have a significant relationship to the beta diversity measure of putative functional capacity (Age: *F* = 0.915, *R* ^2^ = 0.025, *p* = 0.694; Sex: *F* = 0.937, *R* ^2^ = 0.013, *p* = 0.500; Gland: Species: *F* = 0.735, *R* ^2^ = 0.001, *p* = 0.922).

We then tested for differential abundances of metabolic pathways, and to identify differentially abundant pathways between the two gland types in *L. weddelli* and *T. imperator* separately. In both datasets, no pathways were significantly differentially abundant, indicating a similar putative functional capacity in the sternal and suprapubic glands of both species.

## 4 Discussion

We profiled the microbiome present on the surface of two scent glands in two species of wild tamarins and described the presence and abundance of microbial taxa and pathways consistent with those previously associated with VOCs production, offering indirect support for the fermentation hypothesis. Additionally, in *L. weddelli*, we found lower taxonomic diversity and distinct taxonomic composition in suprapubic glands compared to the sternal glands. These findings suggest that species‐specific differences exist in scent gland microbial communities, warranting further investigation to determine the phylogenetic, physiological, ecology and/or behavioural drivers of the variation and the functional consequences.

### 4.1 Taxonomy and Functional Capacity of Glandular Microbiome

Our first aim was to profile the glandular microbiome of two sympatric tamarin species, which revealed a rather homogeneous distribution of taxa, as 77% of species were found in all samples irrespective of species or other demographic variables (Figure 1B). The most dominant bacterial phyla in our study aligned with the bacterial profiles of glandular surfaces or secretions available from other mammals, namely humans, giant pandas ( *Ailuropoda melanoleuca* ), and meerkats ( *Suricata suricatta* ) (Lam et al. 2018; Leclaire et al. 2014; Troccaz et al. 2015; Zhou et al. 2021). While *Firmicutes* often dominate mammalian glandular microbiota (Bowen et al. 2021; Greene et al. 2019; Theis et al. 2013), *Actinomycetota* were the most abundant in our subjects, potentially reflecting their advantage in skin areas rich in sebum, such as the moist and oily scent glands of tamarins, as suggested in humans (Swaney et al. 2023). At the genus level, earlier work identified *Staphylococcus*, *Streptococcus*, and *Corynebacterium* in swab cultures from suprapubic and sternal glands of captive saddleback tamarins ( *Saguinus fuscicollis* ) (Nordstrom et al. 1989). This is partially consistent with our results on *L. weddelli* , where *Staphylococcus* and *Corynebacterium*, but not *Streptococcus*, were among the most abundant genera, whereas these taxa were not dominant in *T. imperator* (Figure 3A). In both tamarin species, abundances of these three genera were lower than in other mammals, where those genera often comprise a quarter or more of the entire community (Callewaert et al. 2013; Li et al. 2016; Rojas et al. 2023; Roze et al. 2010). Our methods, which target bacteria, archaea, eukaryotes, and viruses, showed that each sample hosted a more diverse community than previously found in humans, domestic cats, tamarins, and red foxes (Grice et al. 2008; Nordstrom et al. 1989; Rojas et al. 2023; Ware and Gosden 1980). Interestingly, a significant proportion of the microbes identified in our study consisted of eukaryotes (25%), which are less described than bacteria in scent gland areas of wild mammals. Fungi able to exploit sebum and sweat are increasingly recognized as part of the human skin microbiota (Byrd et al. 2018). Although *Ascomycota* and *Basidiomycota* have often been related to skin diseases in humans (White et al. 2014), both phyla were abundant across all our samples, especially in *T. imperator* , possibly suggesting a nonpathogenic role in these primate taxa.

The microbial metabolic profiles of both our tamarin species were dominated by pathways related to cell metabolism, such as amino acids and nucleotide biosynthesis, and energy metabolism. A previous study on healthy human skin also identified amino acids, lipids, and xenobiotics as the most abundant metabolites, followed by peptides and energy‐related metabolites (Roux et al. 2022). While our results are partially consistent with this, differences in environment and host species should be considered, encouraging studies on the glandular microbiome functions across the primate lineage. For example, xenobiotics found on human skin might originate from external sources like cleaning products, makeup, and other chemicals which are absent in wild tamarins. Notably, both in our samples and in previous studies, amino acid biosynthesis pathways are consistently abundant (James et al. 2013; Lam et al. 2018; Ma et al. 2022; Rojas et al. 2023; Troccaz et al. 2015). In many species of *Staphylococcus*, these pathways are essential for synthesizing proteins, building membranes, and supporting overall metabolism (Roux et al. 2022), and, in the context of our study, they also may be relevant to odor production.

### 4.2 Glandular Microbes That Might Contribute to Odor Production

We found that the scent glands of wild tamarins harbor a microbiome that includes taxa previously reported to produce VOCs (Table 1). This is in line with the predictions of the fermentation hypothesis, which proposes a microbial contribution to individual body odor. Our dataset comprises bacterial taxa known to play a role in body odor in humans, such as *Staphylococcus aureus* , *S. epidermidis* , *S. hominis* , *Corynebacterium tuberculostearicum* , and *C. striatum* (Hara et al. 2014; James et al. 2004; Lam et al. 2018; Troccaz et al. 2015), and species linked to odor production in other mammals, including *Corynebacterium frankenforstense*, *Proteus mirabilis* , *Lactobacillus johnsonii* , *Bacteroides fragilis* , *Staphylococcus* spp., and *Finegoldia magna* (Nordstrom et al. 1989; Rojas et al. 2023; Yamaguchi et al. 2019). These bacterial taxa have been linked to pyruvate and lactate metabolism, as well as branched‐chain and aromatic amino acid metabolism (Hara et al. 2014; Lam et al. 2018), processes that were also prevalent among the microbial metabolic pathways we identified.

Some samples of suprapubic glands of *L. weddelli* had exceptionally high relative abundance of *C. frankenforstense*, hinting it might be a key taxon in suprapubic glands compared to sternal glands (Figures 4A and 5C). Although our data do not allow us to test a causal link between this taxon and VOCs, its role in olfactory communication has already been proposed in a study on domestic cats (Rojas et al. 2023), and our findings further highlight it as a candidate for further investigation in the context of scent production. The second most abundant odor‐related taxon in our dataset was *S. aureus* , which was evenly distributed across individuals. Known for its association with dermatitis and human skin diseases, *S. aureus* is also linked to branched‐chain amino acid metabolism (Roux et al. 2022) and pathways that generate body odor components like diacetyl (Hara et al. 2014). In addition to bacterial taxa, the fungal species *Malassezia furfur* was also abundant in our samples, which has been previously associated with producing VOCs (Gonzalez et al. 2019). The genera *Malassezia* is enriched in lipase genes (Byrd et al. 2018), and likely contributes to lipid synthesis on the skin, a process supported by the presence of lipid‐related pathways in our data.

Interestingly, the superpathways commonly related to odor production, such as leucine and isoleucine biosynthesis, pyruvate fermentation, and amino acid metabolism, were present across gland types and tamarin species, suggesting a shared microbial functional potential that may be relevant to odor release. Altogether, these results depict a functional picture of scent glands in line with other studies in other mammals, laying the groundwork for further investigation into the microbial pathways involved in odor production and the key taxa responsible.

### 4.3 Difference in Diversity and Composition of Microbial Taxa and Metabolic Pathways Between the Two Gland Types

The microbiota of the sternal and suprapubic glands clustered separately, and since, within species, the histology of both glands is similar (Moraes et al. 2006), we suspect that the microbial differences are likely influenced by the distinct roles these glands play in olfactory behaviors. Beta diversity also differed across the three age classes, even though our samples mostly included adults (Table S1). This result is likely driven by the difference between adults and juveniles, which is expected since juveniles only start to develop visible scent glands after puberty, and they rarely perform scent marking (Miller et al. 2003). However, the limited availability of samples from subadults restricts our ability to draw robust conclusions regarding age‐related patterns, calling for future targeted investigations. Nevertheless, in our model, most of the variation was probably due to inter‐individual variation, which is consistent with previous results on other microbiome studies (Asangba et al. 2022; Leclaire et al. 2014).

We did not find support for our prediction that the microbiota of suprapubic glands would be more diverse than that of sternal glands. Instead, sternal gland samples were slightly more diverse. We initially predicted a positive relationship between microbial diversity and frequency of environmental contact during scent marking. However, microbial diversity can be influenced by other factors, such as grooming and social interactions (Sarkar et al. 2020), which may promote more microbial exchange at the level of the sternal gland than in the suprapubic area. Yet, studies on the impact of social behaviors on microbiomes of different body sites remain limited (Björk et al. 2019), which restricts our ability to draw conclusions. Another possible explanation is that the microbiome of suprapubic glands may be more specialized than that of sternal glands. Similar patterns have been observed in other primates: in owl monkeys ( *Aotus nancymaae* ), greater microbial richness correlated with reduced scent marking frequency (Bowen et al. 2021), and in several species of lemurs, dominant males had less diverse sternal gland microbiomes than subordinates, with an enrichment of lactic acid bacteria linked to odor production (Greene et al. 2019). This suggests that selection may favor a microbial community better adapted to odor production in glands frequently involved in olfactory behaviors, such as the suprapubic gland of *L. weddelli* . Importantly, we only sampled the microbiomes on the glandular surface, which provides valuable insights into how external factors and differential gland use may shape microbial communities. However, our sampling methodology did not account for microbes residing deeper within the glands, which may also play a significant role in odor production.

We found partial support for our prediction that the suprapubic glands would have a higher abundance of microbes and metabolic pathways associated with odor production than the sternal ones. While the two gland types of *T. imperator* did not differ in microbial composition, we found 15 taxa reduced in abundance in the sternal glands of *L. weddelli* compared to the suprapubic glands. In general, our analysis found different results between the two tamarin species, while a previous study on the same population had shown that the chemical composition significantly varied between scent gland types in both species (Poirier et al. 2021b). Further cross‐species behavioral research on the differential use of glands in scent marking behavior could help elucidate these findings. Notably, in *L. weddelli* , bacterial taxa of the genera *Corynebacterium* and *Staphylococcus*, known for their association with odor production (James et al. 2004), were less abundant in the sternal glands. Previous research showed that in humans, males with stronger body odor harbored a higher abundance of *Corynebacterium* sp. (Troccaz et al. 2015). These results further support the idea of a more specialized microbial composition in the suprapubic glands of *L. weddelli* , and future studies should explore the potential covariation between microbial communities and VOCs in both species to further investigate this hypothesis. However, we found no significant differences in the abundance of metabolic pathways between the two glands in either tamarin species. This lack of difference may be due to functional redundancy, where multiple microbial taxa may perform similar essential metabolic functions. These results only begin to explore the complex adaptive forces shaping the microbiome of body parts specialized in odor production. Our findings open up intriguing possibilities for a deeper understanding of the proximate mechanisms of olfactory communication and encourage future research into the role of microbial taxa in odor production.

## 5 Limitations of the Study

As with many studies of wild microbiomes, the use of reference databases for taxonomy and functional assignment may introduce bias, as they may not fully capture the diversity of microbial communities present. Furthermore, we chose not to include skin controls from non‐glandular areas because both species engage in scent marking behavior, rubbing their glands on the environment, on existing marks, and on each other (i.e., allomarking) (Heymann 2006). This behavior limits our ability to identify a reliable control site for microbial comparison with skin that is uncontaminated by glandular secretions. Additionally, we inferred the relevance of microbes in odor production based on their metabolic pathways and past research, rather than from chemical data. Although we acknowledge that our study does not establish a direct correlation between microbial communities and odor production, it identifies key taxa and metabolic pathways that warrant further investigation in this context. Finally, our results are cross‐sectional, with one sample per gland per individual, which limits our ability to capture inter‐individual variation over time. Our future research goal is to expand the dataset with multiple years of samples, including individuals from different age classes, and collect chemical data to describe how microbial taxa covary with odor compounds, as well as their metabolic role in odor production. Finally, despite the sample size being relatively small with respect to experimental microbiome studies, our study provides one of the few metagenomic datasets on gland microbiomes generated from wild animals.

## 6 Conclusions

We conclude that scent glands of similar histology harbor different microbiota, and this difference is especially expressed in the species where olfaction is a key sensory system for communication. These findings contribute to the growing body of work suggests that microbial communities may evolve along with hosts as an adaptive trait influencing mammalian socio‐sexual communication. Our study provides a robust metagenomics dataset from wild arboreal mammals, a combination still unique in the field. While the role of bacterial taxa has been previously explored, we provide new insights into the potential complementary role of eukaryotic taxa, which warrants further investigation. By placing this research within a broader selective framework, we can better understand how microbial communities evolve and function in olfaction, ultimately impacting key aspects of host biology, such as reproductive success and social behavior.

## Author Contributions

**Silvia Carboni:** conceptualization (lead), data curation (equal), formal analysis (lead), investigation (equal), methodology (equal), writing – original draft (lead). **Alice C. Poirier:** conceptualization (supporting), data curation (equal), funding acquisition (equal), investigation (equal), methodology (equal), writing – review and editing (equal). **Ana P. Peralta‐Aguilar:** investigation (equal). **Mrinalini Watsa:** funding acquisition (equal), investigation (equal), project administration (equal), writing – review and editing (equal). **Gideon Erkenswick:** funding acquisition (equal), investigation (equal), project administration (equal), writing – review and editing (equal). **Amanda D. Melin:** conceptualization (supporting), data curation (equal), funding acquisition (equal), investigation (equal), methodology (supporting), supervision (lead), writing – review and editing (lead).

## Disclosure

Benefit‐sharing statement: Benefits generated—This research build upon and strengthened an international collaboration by partnering researchers in Canada and the USA with the non‐profit organizations Field Projects International and the In Situ Lab initiative in Peru. These partnerships enabled the collection of samples through coordinated efforts among researchers and contributed to training opportunities in all partner countries. All collaborators are acknowledged as co‐authors on resulting publications, and the data generated have been made publicly available through the database described below. This study further generated novel datasets and scripts that are publically available, as described in the Data Availability statement below.

## Conflicts of Interest

The authors declare no conflicts of interest.

## Supporting information


**Appendix S1:** ece372335‐sup‐0001‐AppendixS1.docx.

## Data Availability

Raw sequence reads are deposited in the NCBI SRA (PRJNA1260568). Sample metadata is stored in the same BioProject, and also provided in the Supporting Information (Table S1). The code for statistical analysis is publicly available on GitHub (https://github.com/silviacarboni/SternalSuprapubicGlandTamarinMicrobiome).
