# Supplementary material for: The Scent Gland Microbiomes of Wild Tamarins Provide New Insight Into Microbial Contributions to Olfactory Communication
Source: Ecol Evol. 2025 Oct 30;15(11):e72335. doi: 10.1002/ece3.72335 (PMC12572820; doi:10.1002/ece3.72335)
Supplement: Supplementary file 1 — Appendix S1: ece372335‐sup‐0001‐AppendixS1.docx. [file ECE3-15-e72335-s001.docx]

**SUPPLEMENTARY MATERIAL**

**Supp. Table 1.** List of the metadata of the 76 samples used in this study. In the table below: SP and ST stand for suprapubic and sternal glands, respectively, LWED for *Leontocebus weddelli* and TIMP for *Tamarinus imperator,* F for female and M for males*.*

| **SampleID** | **GlandType** | **GroupID** | **Species** | **Age** | **Sex** | **Name** |
| --- | --- | --- | --- | --- | --- | --- |
| FPI16206 | SP | AR | LWED | Juvenile | F | AR_2022_4F |
| FPI18946 | ST | AR | LWED | Juvenile | F | AR_2022_4F |
| FPI14707 | SP | AR | LWED | Adult | F | SF1_0F |
| FPI19310 | ST | AR | LWED | Adult | F | SF1_0F |
| FPI14708 | SP | AR | LWED | Juvenile | F | AR_2022_4M_F |
| FPI14636 | ST | AR | LWED | Juvenile | F | AR_2022_4M_F |
| FPI16168 | SP | SS | LWED | Adult | M | SBO |
| FPI18854 | ST | SS | LWED | Adult | M | SBO |
| FPI14656 | SP | SS | LWED | Juvenile | F | SS_2022_4F |
| FPI18855 | ST | SS | LWED | Juvenile | F | SS_2022_4F |
| FPI14680 | SP | SS | LWED | Adult | M | SBW2 |
| FPI19317 | ST | SS | LWED | Adult | M | SBW2 |
| FPI16173 | SP | SS | LWED | Adult | M | SBS |
| FPI18851 | ST | SS | LWED | Adult | M | SBS |
| FPI14652 | SP | SS | LWED | Adult | F | LPG2 |
| FPI18945 | ST | SS | LWED | Adult | F | LPG2 |
| FPI14655 | SP | MI | TIMP | Adult | F | LPR2 |
| FPI18853 | ST | MI | TIMP | Adult | F | LPR2 |
| FPI14653 | SP | MI | TIMP | Adult | M | LBY2 |
| FPI18819 | ST | MI | TIMP | Adult | M | LBY2 |
| FPI16203 | SP | GP | LWED | Adult | M | LBO2 |
| FPI18856 | ST | GP | LWED | Adult | M | LBO2 |
| FPI14702 | SP | GP | LWED | Adult | F | LPW3 |
| FPI18817 | ST | GP | LWED | Adult | F | LPW3 |
| FPI16172 | SP | JI | TIMP | Adult | M | GBR2 |
| FPI18910 | ST | JI | TIMP | Adult | M | GBR2 |
| FPI18906 | SP | IC | TIMP | Adult | F | LPG |
| FPI19309 | ST | IC | TIMP | Adult | F | LPG |
| FPI14682 | SP | IC | TIMP | Subadult | F | J2_F_19 |
| FPI18947 | ST | IC | TIMP | Subadult | F | J2_F_19 |
| FPI14710 | SP | IC | TIMP | Juvenile | M | 4M |
| FPI18911 | ST | IC | TIMP | Juvenile | M | 4M |
| FPI14679 | SP | IC | TIMP | Subadult | M | J2_M_19 |
| FPI18915 | ST | IC | TIMP | Subadult | M | J2_M_19 |
| FPI18907 | SP | OI | TIMP | Adult | M | OBS |
| FPI19313 | ST | OI | TIMP | Adult | M | OBS |
| FPI16204 | SP | OI | TIMP | Adult | F | OPY2 |
| FPI18852 | ST | OI | TIMP | Adult | F | OPY2 |
| FPI16237 | SP | OI | TIMP | Subadult | M | OBG2 |
| FPI18950 | ST | OI | TIMP | Subadult | M | OBG2 |
| FPI16201 | SP | OI | TIMP | Juvenile | M | OI_2022_4M |
| FPI19308 | ST | OI | TIMP | Juvenile | M | OI_2022_4M |
| FPI16240 | SP | WI | TIMP | Adult | M | WBG2 |
| FPI18913 | ST | WI | TIMP | Adult | M | WBG2 |
| FPI18909 | SP | WI | TIMP | Adult | M | WBO |
| FPI18949 | ST | WI | TIMP | Adult | M | WBO |
| FPI18878 | SP | YM | TIMP | Juvenile | F | YM_2022_4F |
| FPI19315 | ST | YM | TIMP | Juvenile | F | YM_2022_4F |
| FPI16239 | SP | YM | TIMP | Juvenile | M | YM_2022_4M |
| FPI18849 | ST | YM | TIMP | Juvenile | M | YM_2022_4M |
| FPI18813 | SP | S7 | LWED | Adult | M | YBS2 |
| FPI18857 | ST | S7 | LWED | Adult | M | YBS2 |
| FPI18812 | SP | S7 | LWED | Adult | F | YPL2 |
| FPI18821 | ST | S7 | LWED | Adult | F | YPL2 |
| FPI18816 | SP | S7 | LWED | Juvenile | M | S7_2022_5M |
| FPI19318 | ST | S7 | LWED | Juvenile | M | S7_2022_5M |
| FPI18815 | SP | S7 | LWED | Adult | M | YBG |
| FPI18818 | ST | S7 | LWED | Adult | M | YBG |
| FPI18942 | SP | WF | LWED | Adult | M | WBR2 |
| FPI19316 | ST | WF | LWED | Adult | M | WBR2 |
| FPI14658 | SP | AR | LWED | Adult | M | OBL |
| FPI19312 | ST | AR | LWED | Adult | M | OBL |
| FPI14681 | SP | SS | LWED | Juvenile | M | SS_2022_4M |
| FPI18850 | ST | SS | LWED | Juvenile | M | SS_2022_4M |
| FPI16171 | SP | GP | LWED | Adult | M | LBBl |
| FPI18914 | ST | GP | LWED | Adult | M | LBBl |
| FPI14704 | SP | MI | TIMP | Adult | M | LBO2 |
| FPI18951 | ST | MI | TIMP | Adult | M | LBO2 |
| FPI14709 | SP | JI | TIMP | Adult | F | JI_2022_RC |
| FPI18948 | ST | JI | TIMP | Adult | F | JI_2022_RC |
| FPI18879 | SP | YM | TIMP | Adult | M | YBS3 |
| FPI18820 | ST | YM | TIMP | Adult | M | YBS3 |
| FPI16169 | SP | JI | TIMP | Adult | M | RBW |
| FPI18912 | ST | JI | TIMP | Adult | M | RBW |
| FPI14706 | SP | GD | LWED | Adult | F | GD_2022_0F |
| FPI16207 | SP | IC | TIMP | Adult | M | GBG |

**Supp. Table 2.** Results of AICc tests comparing null models to the model including the type of gland as a fixed effect. The response variable is either the Shannon or the Chao1 index calculated on the abundances of taxa or metabolic pathways in our microbial data. For each model pair, the best one is listed as first.

| Data type | Diversity measure | Model | Log Likelihood | AICc | ΔAICc | Weight |
| --- | --- | --- | --- | --- | --- | --- |
| Taxonomy | Shannon | ~ Gland * Species +  (1 \| GroupID/Name) | -42.883 | 101.4 | 0.00 | 0.691 |
|  |  | ~ Gland * Species + Age +  (1 \| GroupID/Name) | -42.710 | 103.6 | 2.16 | 0.235 |
|  |  | ~ Gland * Species + Sex +  (1 \| GroupID/Name) | -42.579 | 105.9 | 4.47 | 0.074 |
|  |  | ~ 1 +  (1 \| GroupID/Name) | -55.059 | 118.7 | 17.27 | 0.00 |
|  | Chao1 | ~ 1 +  (1 \| GroupID/Name) | -502.932 | 1014 | 0.00 | 0.662 |
|  |  | ~ Gland * Species +  (1 \| GroupID/Name) | -500.409 | 1016 | 2.04 | 0.239 |
|  |  | ~ Gland * Species + Age +  (1 \| GroupID/Name) | -500.358 | 1019 | 4.44 | 0.072 |
|  |  | ~ Gland * Species + Sex +  (1 \| GroupID/Name) | -500.038 | 1021 | 6.38 | 0.027 |
| Functional capacity | Shannon | ~ 1 +  (1 \| GroupID/Name) | 68.046 | -127.5 | 0.00 | 0.915 |
|  |  | ~ Gland * Species +  (1 \| GroupID/Name) | 68.894 | -122.1 | 5.39 | 0.062 |
|  |  | ~ Gland * Species + Sex +  (1 \| GroupID/Name) | 68.943 | -119.7 | 7.79 | 0.019 |
|  |  | ~ Gland * Species + Age +  (1 \| GroupID/Name) | 68.915 | -117.1 | 10.43 | 0.005 |
|  | Richness | ~ 1 +  (1 \| GroupID/Name) | **-**416.020 | 840.6 | 0.00 | 0.958 |
|  |  | ~ Gland * Species +  (1 \| GroupID/Name) | -415.951 | 847.5 | 6.95 | 0.030 |
|  |  | ~ Gland * Species + Sex +  (1 \| GroupID/Name) | -415.874 | 849.9 | 9.29 | 0.009 |
|  |  | ~ Gland * Species + Age +  (1 \| GroupID/Name) | -415.766 | 852.3 | 11.66 | 0.003 |

**Supp. Table 3.** Measures of length and width (mm) of the suprapubic (SP) and sternal (ST) glands for the two tamarin species. For each individual we divided the length and width of each gland by its body weight (BW in g), then we calculated the mean across all samples of the same gland and tamarin species.

| Species | Number of samples | Gland Type | Mean(L/BW) mm/g | | Mean(W/BW) mm/g | |
| --- | --- | --- | --- | --- | --- | --- |
| *Leontocebus weddelli* | 13 | SP | 0.051 | | 0.026 | |
|  | 8 | ST | 0.020 | | 0.017 | |
| *Tamarinus imperator* | 11 | SP | | 0.039 | | 0.018 |
|  | 12 | ST | | 0.020 | | 0.014 |

**Supp. Table 4.** Results of the ANCOM-BC tests on the differential abundance of microbial species, between suprapubic (n = 19) and sternal glands (n = 18) of *L. weddelli* (adjusted p-value < 0.01). For each taxon, the table reports the values of log fold changes, W values and adjusted p-values for the gland comparison, with sternal gland being the reference category.

| Microbial taxa | **LogFold Change** | W values | adjusted p-values |
| --- | --- | --- | --- |
| *Corynebacterium atypicum* | -3.958 | -7.203 | 5.072e-04 |
| *Staphylococcus auricularis* | -3.655 | -7.855 | 7.558e-05 |
| *Corynebacterium frankenforstense* | -3.568 | -6.693 | 2.318e-03 |
| *Staphylococcus sp. ACRSN* | -3.359 | -8.820 | 6.362e-06 |
| *Corynebacterium uberis* | -3.283 | -7.478 | 2.710e-04 |
| *Corynebacterium renale* | -2.745 | -6.601 | 3.051e-03 |
| *Staphylococcus intermedius* | -2.477 | -6.854 | 1.659e-03 |
| *Geobacillus sp. FJAT-46040* | -2.333 | -8.605 | 8.409e-03 |
| *Corynebacterium lubricantis* | -2.124 | -6.335 | 6.781e-03 |
| *Corynebacterium uterequi* | -2.046 | -6.327 | 6.952e-03 |
| *Parabacteroides sp. HGS0025* | -2.017 | -6.460 | 6.072e-03 |
| *Collinsella sp. AF05-8-2* | -1.956 | -7.137 | 6.956e-03 |
| *Corynebacterium callunae* | -1.858 | -6.295 | 7.670e-03 |
| *Caniella muris* | -1.410 | -6.517 | 3.922e-03 |
| *Tissierella creatinophila* | -1.149 | -6.427 | 5.143e-03 |


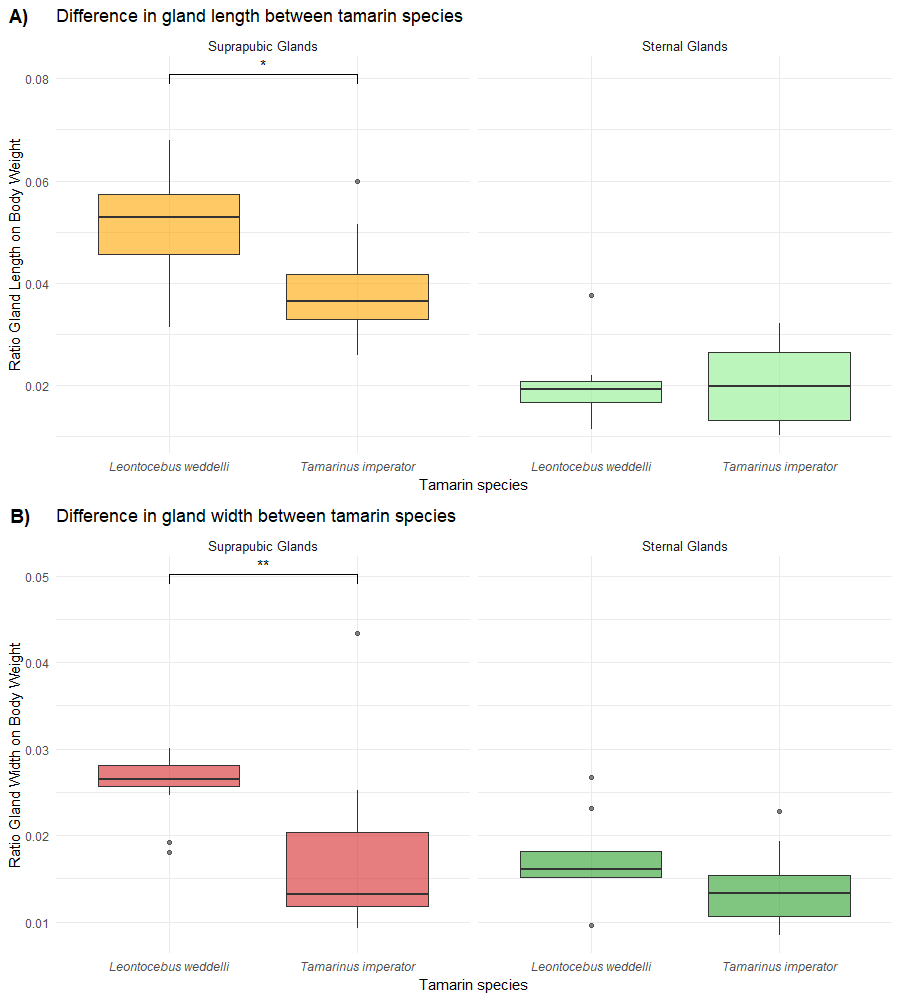


**Supp. Figure 1.** Boxplot representation of the differences in morphology of suprapubic or sternal glands between *L. weddelli* and *T. imperator.* Panel **A)** shows the distribution of the ratio of gland length to body weight (mm/g) for each individual, while panel **B)** shows the distribution of the ratio of gland width to body weight (mm/g) for each individual. Only the interspecies differences in suprapubic glands were significant, with larger glands relative to body weight in *L. weddelli* compared to *T. imperator*. The * symbols above the boxplots indicate statistical significance (*: p < 0.05; **: p< 0.01)


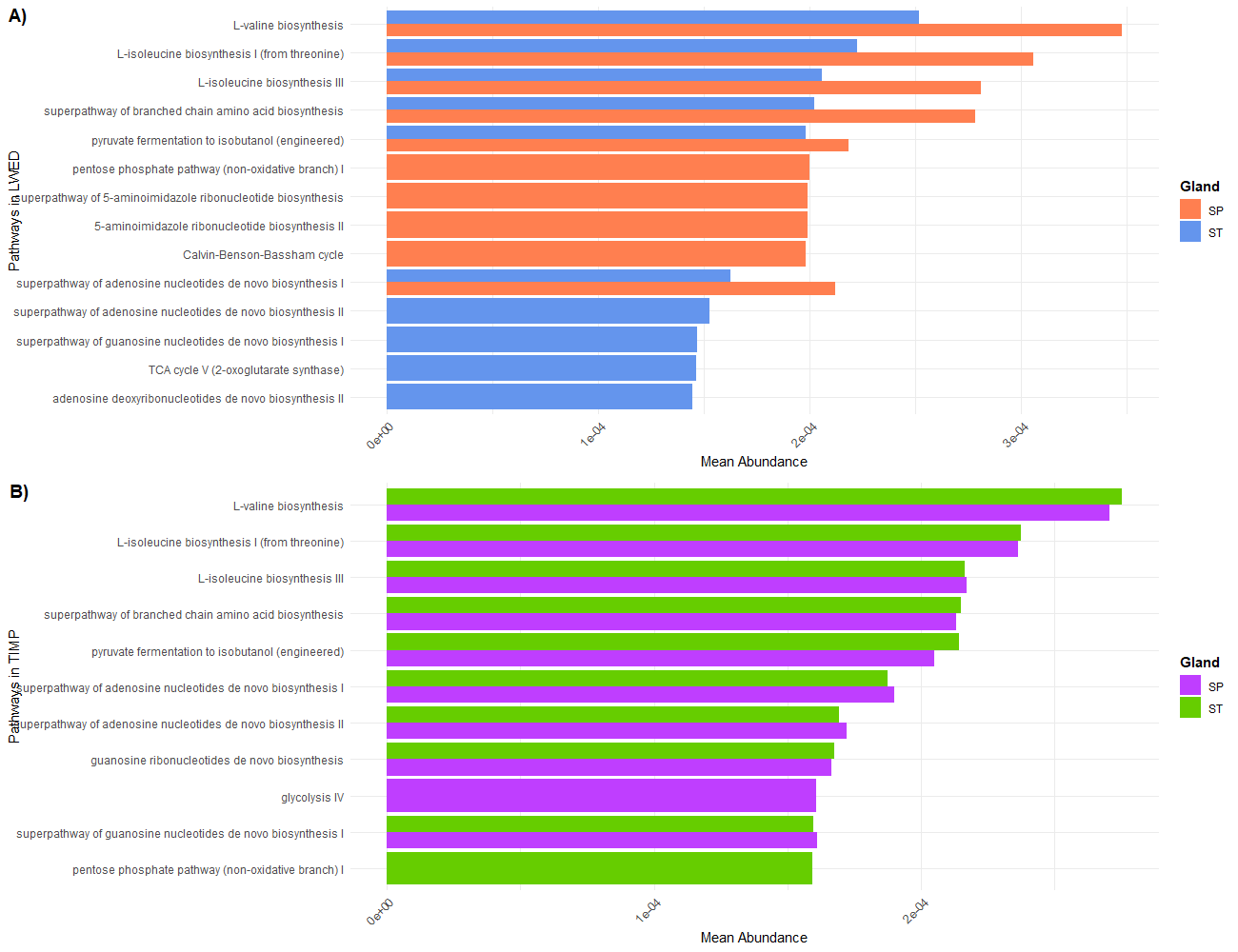
**Supp. Figure 2.** Most abundant metabolic pathways in the suprapubic (SP) and sternal (ST) glands of *L. weddelli* (LWED) and *T. imperator* (TIMP). **A)** Metabolic pathways in *L. weddelli*, showing the mean abundance of each of the 10 most abundant pathways in the suprapubic and sternal glands. **B)** Metabolic pathways in *T. imperator*, with similar comparisons of the suprapubic and sternal glands.


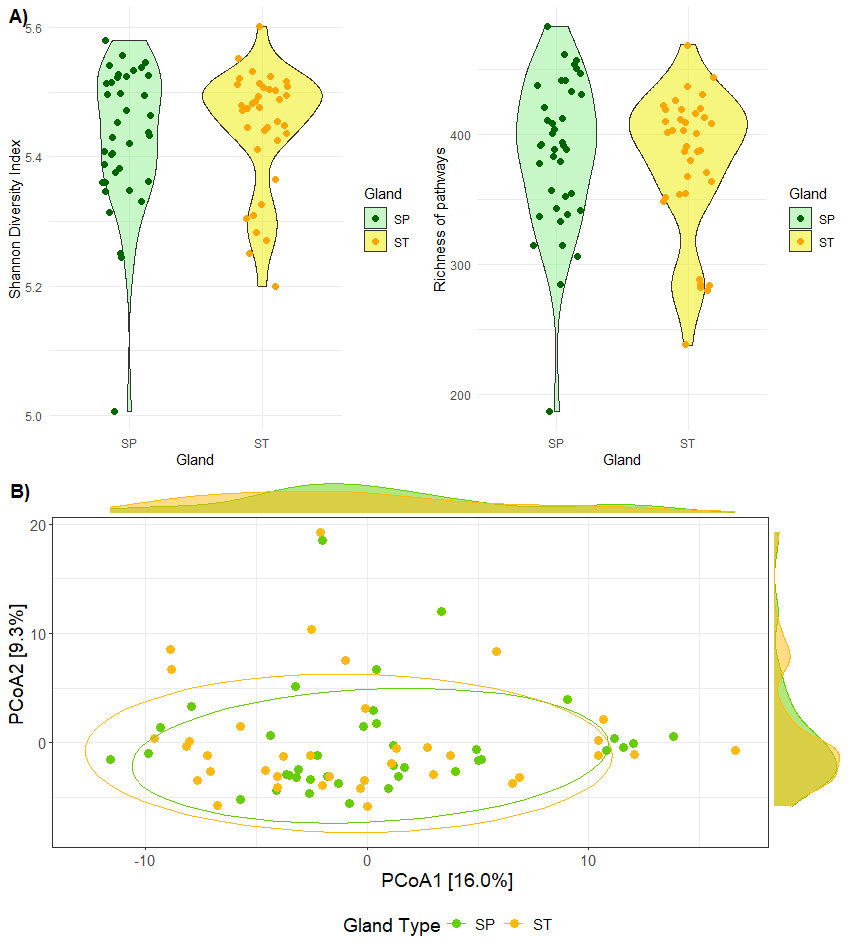


**Supp. Figure 3.** Alpha and beta diversity measures for the metabolic pathways in the suprapubic and sternal gland samples of both tamarin species. **A)** Neither of the Shannon index nor the richness of metabolic pathways showed a difference between the two glands. **B)** Principal Coordinates Analysis (PCoA) calculated using the robust Aitchison distance, which showed that the x-axis (PCoA1) and y-axis (PCoA2) explain 16% and 9.3% of the variation in microbial community composition, respectively. The ellipses represent the 85% confidence interval for each gland type. providing an indication of the clustering and dispersion of samples within each group. The density plots on the axes represent the microbial communities across the gland types.
